# Polyphenols Targeting Brain Cells Longevity, Brain's Redox Status, and Neurodegenerative Diseases

**DOI:** 10.1155/2018/7402795

**Published:** 2018-08-09

**Authors:** Anat Elmann, Chin-Kun Wang, David Vauzour

**Affiliations:** ^1^Department of Food Quality and Safety, Agricultural Research Organization, Volcani Center, POB 15159, Rishon LeZion 7528809, Israel; ^2^School of Nutrition, Chung Shan Medical University, Taichung City, Taiwan; ^3^Norwich Medical School, Faculty of Medicine and Health Sciences, University of East Anglia, Norwich NR4 7UQ, UK

It is becoming widely accepted that polyphenols may act as strong dietary strategies to combat neurodegenerative diseases. The articles in this special issue include both basic scientific studies along with review articles focused on demonstrating and understanding the underlying mechanisms by which purified polyphenols and/or polyphenols containing plant extracts affect cultured brain cells, animal models of Parkinson's or Alzheimer's diseases, and depression.

Alzheimer's disease is a proteinopathy characterised by the accumulation of hyperphosphorylated Tau and *β*-amyloid. Autophagy is a physiological process by which aggregated proteins and damaged organelles are eliminated through lysosomal digestion. Autophagy deficiency has been demonstrated in Alzheimer's patients. In the research article “Benefit of Oleuropein Aglycone for Alzheimer's Disease by Promoting Autophagy” by J. G. Cordero et al., the authors demonstrated that oleuropein aglycone, present in high concentration in extra virgin olive oil, is capable of inducing autophagy in both *in vitro* and *in vivo* models, and that this leads to an improvement in cognitive impairment as well as in *β*-amyloid and Tau aggregation. The authors propose that supplementation of diet with extra virgin olive oil may have potential benefits for Alzheimer's disease patients through the induction of autophagy by oleuropein aglycone.

The aim of the paper entitled “Mangiferin and Morin Attenuate Oxidative Stress, Mitochondrial Dysfunction, and Neurocytotoxicity, Induced by Amyloid Beta Oligomers” by E. Alberdi et al. was to investigate the neuroprotective effects of the polyphenols morin and mangiferin against A*β* oligomers and their mechanisms of action. The authors found that these polyphenols mitigated the mitochondrial dysfunction by mechanisms that regulated mitochondrial calcium homeostasis, mitochondrial membrane potential, and release to cytosol of proapoptotic cytochrome c. Moreover, morin and mangiferin treatments restored the altered redox homeostasis of antioxidant enzymes in neurons treated with oligomeric A*β*. Consequently, these polyphenols reduced protein oxidation and reestablished bioenergetic failure in A*β*-treated neurons, contributing to the substantial reduction of neuronal death.

Chlorogenic acid is a plant polyphenol, which was found to be a major bioactive constituent of *Dendropanax morbiferus* leaves extract. The contribution by S.-Y. Park et al. entitled “Aqueous Extract of *Dendropanax morbiferus* Leaves Effectively Alleviated Neuroinflammation and Behavioral Impediments in MPTP-Induced Parkinson's Mouse Model” evaluated the underlying molecular mechanism of the antineuroinflammatory activity and the neuroprotective potential of *Dendropanax morbiferus* leaves and its bioactive compound chlorogenic acid in *in vitro* and *in vivo* experimental models of Parkinson's disease. The authors demonstrate that prophylactic treatment of *Dendropanax morbiferus* leaves improved the behavioral deficits, inhibited the microglial-mediated neuroinflammation, and protected dopaminergic neuronal loss by restoring tyrosine hydroxylase levels in brain tissues of the MPTP-induced Parkinson's mouse model. Further, polyphenols that affect Alzheimer's and Parkinson's diseases were also summarised in the systematic review article entitled “Flavonoids as Therapeutic Agents in Alzheimer's and Parkinson's Diseases: A Systematic Review of Preclinical Evidences” by R. B. de Andrade Teles et al. The results of their survey showed that flavonoids, which are the major group of polyphenols, are promising candidates for drug development, but there is a lack of translational research and clinical evidences which might facilitate their development as drugs.

Polyphenols also received growing interest due to their potential benefits in treating psychiatric disorders. Their antioxidant and anti-inflammatory activities as well as their ability to modulate synaptic plasticity contribute to their mechanism of action. The paper by J. Wang et al. entitled “An Extract of *Artemisia dracunculus* L. Promotes Psychological Resilience in a Mouse Model of Depression” aims at investigating the potential therapeutic value of this botanical extract in a model of depression. *Artemisia dracunculus* L. (Russian tarragon) extract contains flavonoids, coumarins, and phenylpropanoid acids. Using a repeated social defeat stress (RSDS) model of depression, the authors demonstrate that oral administration of *Artemisia dracunculus* L. extract promotes resilience to RSDS-mediated depression-like phenotypes. The authors also show that the behavioral improvements are associated with attenuation of stress-mediated induction of inflammatory cytokines in the periphery and alteration of synaptic plasticity in the nucleus accumbens.

We hope that this special issue would stimulate scientists from the agricultural, plant sciences, and the medical communities to promote interdisciplinary studies that will lead to the development of polyphenols as drugs or food supplements for neurodegenerative diseases.

